# Methyltransferase-like 3 Aggravates HCC Development via Mediating N6-Methyladenosine of Ubiquitin-Specific Protease 7

**DOI:** 10.1155/2022/6167832

**Published:** 2022-05-05

**Authors:** Daiyue Yuan, Jie Chen, Qingya Hao, Peng Zhang, Zhong Chen

**Affiliations:** ^1^Department of Hepatobiliary Surgery, The First Affiliated Hospital of Soochow University, Suzhou 215006 ., China; ^2^Department of Hepatobiliary Surgery, The Second Affiliated Hospital of Nantong University, Nantong 226001, China; ^3^Department of General Surgery, Affiliated Hospital of Nantong University, Nantong 226001, China

## Abstract

We aimed to investigate the role of methyltransferase-like 3 (METTL3) in regulating HCC by mediating m6A level of ubiquitin-specific protease (USP7). METTL3 levels and m6A contents in HCC tissues and cells were detected. Potential correlations between METTL3 level and lymphatic metastasis, tumor size, tumor staging, and overall survival of HCC patients were analyzed. Moreover, its regulatory effects on proliferative, migratory, and invasive rates of HCC cells were examined. Potential methylation of USP7 in HCC was predicted using an online software, and the correlation between USP7 and METTL3 was assessed. METTL3 and m6A were increased both in HCC cells and tissues. High level of METTL3 was associated with the incidence of lymphatic metastasis, large tumor size, advanced tumor staging, and low overall survival of HCC. Silencing of METTL3 reduced proliferation, migration, and invasion rates. USP7 was predicted to have a methylation site regulated by METTL3. It was upregulated in HCC and associated with METTL3 level positively. USP7 silencing decreased proliferation, migration, and invasion rates of HCC cells. METTL3 promotes HCC to proliferate, migrate, and invade by regulating m6A methylation of USP7.

## 1. Introduction

The incidence and mortality of liver cancer rank fifth and third, respectively [1–3]. Hepatocellular carcinoma (HCC) is one of the main three subtypes of liver cancer; it accounts for 90% of all reported cases [4]. Typical symptoms of HCC in the early stage are lacked, leading to a high diagnostic rate of advanced HCC. At present, liver transplantation is the only way that is possible to cure HCC. However, the source of liver transplantation is extremely rare. Although surgical resection of HCC is also effective, a high postoperative recurrence and a small range of apply crowd significantly restricts the clinical application of surgery [5]. About 80-90% of HCC patients have lost the optimal opportunity of surgical resection because of the already developed metastases [6]. Moreover, HCC is insensitive to chemotherapy and radiotherapy. The prognosis of middle-stage and advanced HCC is extremely poor since therapeutic options are limited. It is urgent to clarify the pathogenesis and molecular pathway of HCC, which is beneficial to improve the prognosis.

Methylation of RNA mainly includes m1A (N1-methyladenosine), m6A (N6-methyladenosine), and m5C (C5-methylcytidine) [7]. Among them, m6A is widely distributed in 7,000 mRNAs and 300 ncRNAs, serving as the most abundant RNA modification of eukaryotes [8–11].

The dynamic reversible process of m6A methylation has been clearly revealed, involving the writer, reader, and eraser [8]. m6A methyltransferases (writers) establish a complex constituted by METTL3, METTL14, and WTAP, which is responsible for writing methylation information into RNAs [12, 13]. Demethylases ALKBH5 and FTO (erasers) are used to reverse or eliminate the process of RNA modification [14–16]. m6A participates in every step of RNA metabolism, which affects RNA stability, translation efficiency, alternative splicing, and positioning. Any abnormality of m6A would cause human diseases like tumors, neurological diseases, metabolic diseases, and embryos developmental delay.

Methyltransferase-like 3 induces the writing process of m6A methylation and exerts either a carcinogenic or anticancer effect and is closely related to poor prognosis [17–20]. Interestingly, the role of METTL3 differs in different types of tumors. Several studies reported that that METTL3 is upregulated in many cancer types including adenocarcinoma of lung that stimulates the growth of cancer cells [21]. METTL3 a potential oncogene exaggerates several cancers progression, including hematopoietic malignancies. METTL3 expressions were reported to be responsible indicator of tumor microenvironment and were used to predict the prognosis of pancreatic cancer patients [22]. METTL3 is also upregulated in glioblastoma, and silence of METTL3 inhibits the growth of glioma stem cells by downregulating POU3F2, SOX2, SALL2, OLIG2, and other glioma recombinant factors [23]. In our current study, we explored the biofunctions of METTL3-induced m6A methylation in the development of HCC.

## 2. Materials and Methods

### 2.1. Collection of HCC Tissues

Fifty HCC tissues and paired normal ones were collected from HCC patients treated in the First Affiliated Hospital of Soochow University from October 2018 to March 2019. Tumor staging was assessed by the guideline proposed by UICC (Union for International Cancer Control). The Ethics Committee of The First Affiliated Hospital of Soochow University approved this study (No.201784). We got written informed consent from the participants before the study.

### 2.2. Cell Transfection

HCC cells (HCCLM3, MHCC97-L, Hep3B, and Huh7) and L02 cell lines (human normal liver cell line) purchased from ATCC (Manassas, VA, USA) were used in this study. METTL3 siRNA, USP7 (ubiquitin-specific protease) siRNA, or negative control together with Lipofectamine 2000 (Beyotime, Shanghai, China) were used for the cell transfection, followed by qRT-PCR for the transfection efficacy determination.

### 2.3. qRT-PCR

The total RNAs extracted using TRIzol (Beyotime, Shanghai, China) were reversely transcribed into cDNAs followed by qPCR according to the manufacturer's protocols. Sequences of primers used were shown as follows: METTL3 (F: 5′-CTCTGGGGGTATGAACGGG-3′, R: 5′-CTCTGGGGGTATGAACGGG-3′); USP7 (F: 5′-CCCTCCGTGTTTTGTGCGA-3′, R: 5′-AGACCATGACGTGGAATCAGA-3′); GAPDH (F: 5′-GGAATCCACTGGCGTCTTCA-3′, R: 5′-GGTTCACGCCCATCACAAAC-3′).

### 2.4. Detection of m6A Methylation

RNA m6A was measured using EpiQuik m6A RNA Methylation Kit according to the manufacturer's protocols by detecting 450 nm absorbance.

### 2.5. Cell Counting Kit-8 (CCK-8)

Cells were seeded in a 96-well plate with 2 × 10^3^ cells/well. After cell adherence, cells were treated with CCK-8 solution (10 *μ*L) (Beyotime, Shanghai, China). Following 2-h cell culture, the absorbance at 450 nm was detected.

### 2.6. Western Blot

Protein extracted were separated and then loaded on PVDF filter membranes. After the electrophoresis, the membranes were incubated with primary antibodies (GAPDH: cat#AF5009, METTL3: cat#ab195352, and USP7: cat#ab108931) (Abcam, Cambridge, MA, USA). Finally, the bands were further exposed via ECL kit (Beyotime, Shanghai, China) after incubation with secondary antibodies for 2 h.

### 2.7. Statistical Analysis

SPSS statistical analysis software (version 26.0, IBM, Armonk, NY, USA) was used for data analysis. Comparisons between groups were performed using independent *t* tests or one-way ANOVA test followed by least significant difference as its post hoc test. Statistical significance was set as *p* < 0.05.

## 3. Results

### 3.1. Upregulated METTL3 in HCC

**Figure 1:**
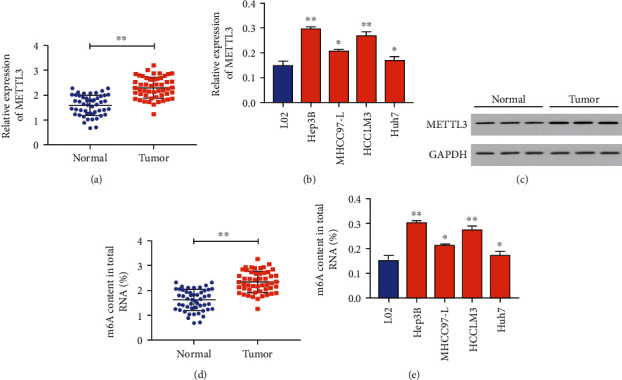
Upregulated METTL3 in hepatocellular carcinoma. (a) METTL3 expressions in liver cancer tissues and adjacent normal ones; (b) METTL3 expressions in HCC cell lines and the normal liver cell line; (c) protein level of METTL3 in 3 pairs of HCC tissues and adjacent normal ones; (d) the m6A content in total RNA of HCC tissues and adjacent normal ones; (e) m6A content of total RNA in HCC cell lines and the normal liver cell line (∗*p* < 0.05 and ∗∗*p* < 0.01).

Compared with adjacent tissues, METTL3 was higher in HCC tissues (Figures 1(a) and 1(c)). As expected, it was also upregulated in HCC cells (Figure 1(b)). The m6A level was much higher in HCC tissues and cells as well (Figures 1(d) and 1(e)).

### 3.2. METTL3 Predicted a Poor Prognosis of HCC

**Figure 2:**
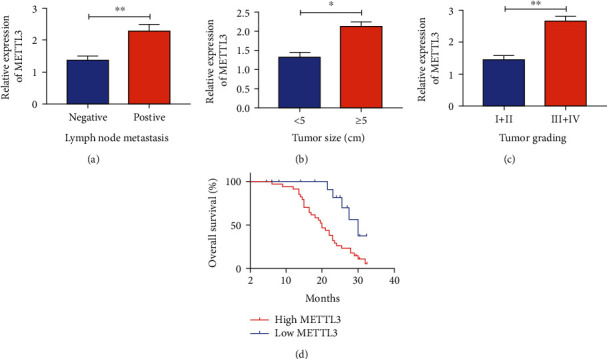
METTL3 was unfavorable to the prognosis of HCC. (a) Relative level of METTL3 in HCC patients with negative or positive lymphatic metastasis; (b) METTL3 expression in HCC patients with different tumor sizes; (c) relative level of METTL3 in HCC patients at different stages; (d) overall survival of HCC patients in high- or low-METTL3 groups (∗*p* < 0.05 and ∗∗*p* < 0.01).

By analyzing clinical data of recruited HCC, higher METTL3 was detected in patients with lymphatic metastasis (Figure 2(a)), ≥5 cm of tumor size (Figure 2(b)), or stage III + IV (Figure 2(c)). Moreover, we analyzed follow-up data of them and found that a high level of METTL3 was negatively correlated with the overall survival of HCC patients (Figure 2(d)). It is concluded that METTL3 may be a promising biomarker predicting the poor prognosis of HCC.

### 3.3. METTL3 Promoted HCC Cells to Proliferate, Migrate, and Invade

**Figure 3:**
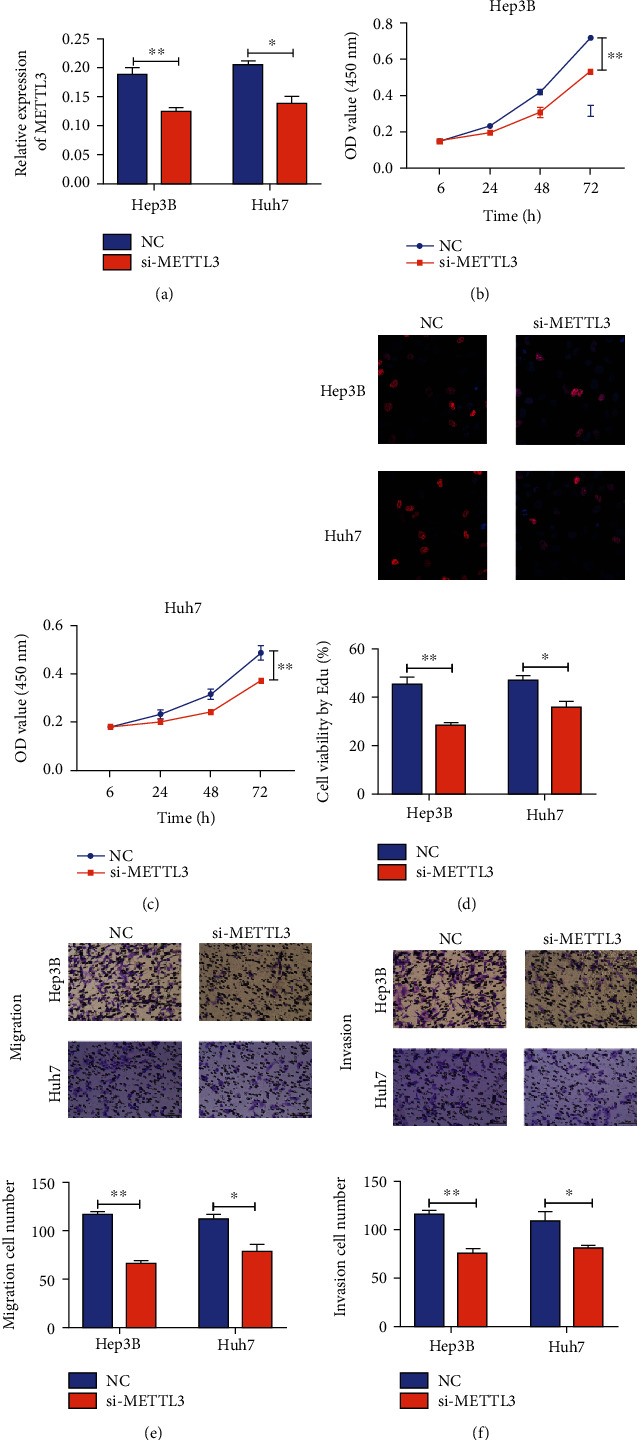
METTL3 promoted HCC cells to proliferate, migrate, and invade. (a) Relative level of METTL3 in Hep3B and Huh7 cells in different groups; cell viability in Hep3B (b) and Huh7 cells (c) transfected with NC or si-METTL3 at 6, 24, 48, and 72 h; (d) Edu assay for Hep3B and Huh7 cells in different groups; (e) migratory cell number in Hep3B and Huh7 cells in different groups; (f) invasive cell number in Hep3B and Huh7 cells. (∗*p* < 0.05 and ∗∗*p* < 0.01).

Hep3B and Huh7 cells were used for exploring the potential underlying mechanism. We first examined that transfection of si-METTL3 obviously downregulated METTL3 (Figure 3(a)). Knockdown of METTL3 markedly decreased cell viability (Figures 3(b) and 3(c)) and EdU-positive cell number (Figure 3(d)), indicating the suppressed proliferative capacity. Besides, relative numbers of migratory and invasive cells were declined by transfection of si-METTL3, suggesting the inhibited migratory and invasive capacities of HCC (Figures 3(e) and 3(f)).

### 3.4. METTL3 Regulated USP7 by m6A Methylation

**Figure 4:**
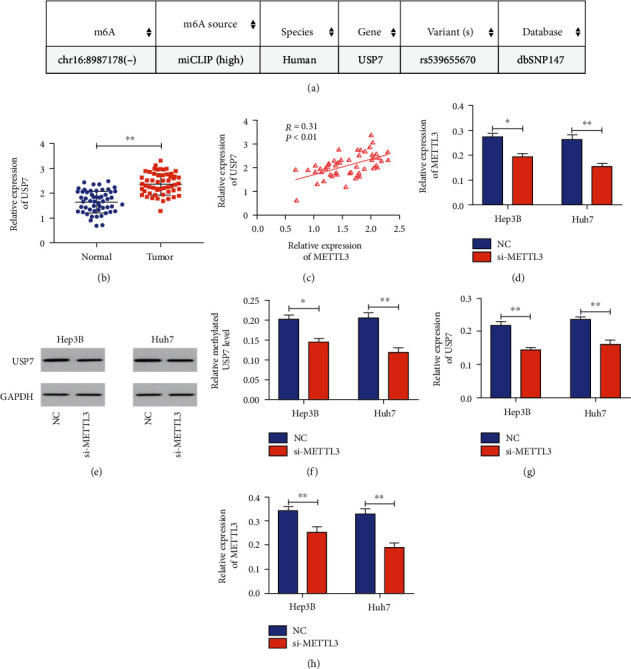
METTL3 regulated USP7 by m6A methylation. (a) Prediction of m6A methylation in the USP7 gene; (b) USP7 expression in HCC tissues and adjacent normal ones; (c) METTL3 was positively correlated with USP7 in HCC tissues; (d) relative level of USP7 in Hep3B and Huh7 cells in different groups; (e) protein level of USP7 in Hep3B and Huh7 cells; (f) relative methylated level of USP7 in Hep3B and Huh7 cells in different groups; (g) USP7 expression in Hep3B and Huh7 cells in different groups; (h) METTL3 expression in Hep3B and Huh7 cells (∗*p* < 0.05 and ∗∗*p* < 0.01).

m6A methylation in the USP7 gene was predicted online (Figure 4(a)). Hence, we speculated that METTL3 may be able to regulate USP7 level. USP7 was increased in HCC tissues (Figure 4(b)) and associated with METTL3 level positively (Figure 4(c)). Knockdown of METTL3 decreased USP7 at both mRNA and protein levels in HCC cells (Figures 4(d) and 4(e)), further confirming their positive correlation. Methylated level of USP7 was markedly reduced by knockdown of METTL3 (Figure 4(f)). As expected, knockdown of USP7 could downregulate METTL3 in HCC cells (Figures 4(g) and 4(h)). To sum up, relative level of USP7 in HCC cells could be positively regulated by METTL3 through m6A methylation.

### 3.5. USP7 Promoted HCC Cells to Proliferate, Migrate, and Invade

**Figure 5:**
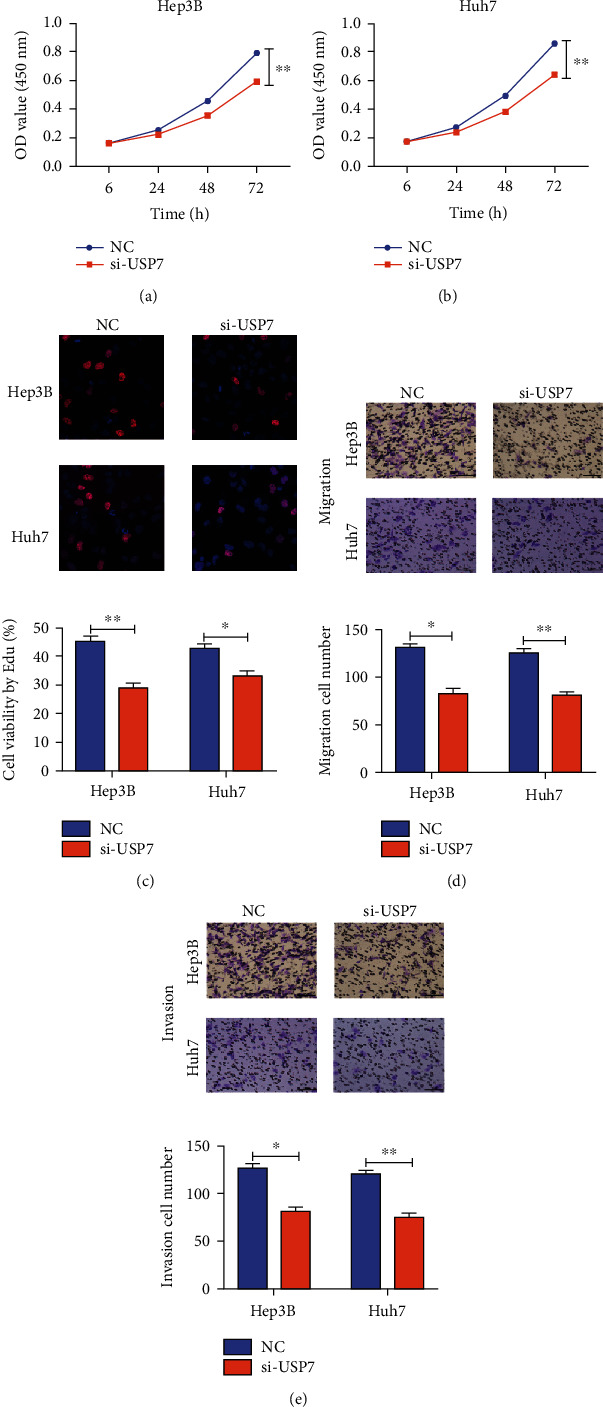
USP7 promoted HCC cells to proliferate, migrate, and invade. (a, b) Cell viability in Hep3B and Huh7 cells in different groups at 6, 24, 48, and 72 h; (c) Edu assay for Hep3B and Huh7 cells in different groups; (d) migratory cell number in Hep3B and Huh7 cells in different groups; (e) invasive cell number in Hep3B and Huh7 (∗*p* < 0.05 and ∗∗*p* < 0.01).

Potential influences of USP7 on cellular functions of HCC were finally explored. Proliferative rate of HCC cells was markedly declined by transfection of si-USP7 (Figures 5(a) and 5(c)). In addition, migratory and invasive capacities were inhibited in HCC cells with USP7 knockdown (Figures 5(d) and 5(e)). It is indicated that USP7 promoted HCC cells to proliferate, migrate, and invade.

## 4. Discussion

Conventional therapies of liver cancer are largely restricted by the low rate of surgical resection, high rate of postoperative recurrence, low response rate to chemotherapy and/or radiotherapy, and rare source of liver transplantation. In recent years, molecular targeted therapy has been well concerned [24]. Sorafenib was currently approved by FDA for medical use of advanced liver cancer. However, Sorafenib does not directly target cancer cells but aims to against angiogenesis by targeting endothelial cells [25]. As a result, the anticancer efficacy of Sorafenib is not satisfactory. Clarifying the exact pathogenesis of HCC is therefore beneficial to develop novel therapeutic targets.

USP7 is a conserved protein initially isolated in Vmw110. It is essential to multiple life activities [26]. USP7 is a vital regulator involved in the antiviral pathway, although its mechanism is unclear [27, 28]. Ubiquitination and deubiquitination exert critical functions in the development of liver diseases. USP7 directly regulates the fate of some substrates including proteasome degradation, and it is mainly responsible for mediating cell cycle progression [29, 30]. USP7 inhibitors are potential therapeutic targets for inducing cancer cell apoptosis. It has been reported that more than a hundred deubiquitinating enzymes were reported as ubiquitin-specific protease 7 (USP7), which reportedly belongs to the largest subfamily of proteases [31]. USP7 reported to have a potential role in regulation of tumor suppressor gene p53 and its E3 ubiquitin ligase and mouse double minute 2 homolog (MDM2), as well as several proteins important to cell cycle [32, 33].

Previous evidence showed that USP7 was increased in HBV-related liver cancer [34]. We believed that USP7 is of significance in the development of HCC.

Our findings showed that METTL3 was upregulated in HCC. Higher METTL3 indicated high incidence of lymphatic metastasis, large tumor size, advanced tumor staging, and low overall survival of HCC. As predicted in bioinformatic website, m6A methylation was identified in the USP7 gene. Through a series of assays, we proved that METTL3 promoted proliferative, migrative, and invasive abilities of HCC cells by regulating m6A methylation of USP7, thus aggravating of HCC development.

## 5. Conclusion

Silencing of METTL3 reduced proliferation, migration, and invasion rates. USP7 was predicted to have a methylation site regulated by METTL3. It was upregulated in HCC and associated with METTL3 level positively. USP7 silencing decreased proliferation, migration, and invasion rates of HCC cells. METTL3 promotes HCC to proliferate, migrate, and invade by regulating m6A methylation of USP7.

## Data Availability

The datasets used and analyzed during the current study are available from the corresponding author on reasonable request.
